# From Physiology to Pathology of Astrocytes: Highlighting Their Potential as Therapeutic Targets for CNS Injury

**DOI:** 10.1007/s12264-024-01258-3

**Published:** 2024-07-30

**Authors:** Yimin Yuan, Hong Liu, Ziwei Dai, Cheng He, Shangyao Qin, Zhida Su

**Affiliations:** 1 Institute of Neuroscience, Key Laboratory of Molecular Neurobiology of Ministry of Education and the Collaborative Innovation Center for Brain Science, Naval Medical University, Shanghai, 200433 China; 2 Department of Pain Medicine, School of Anesthesiology, Naval Medical University, Shanghai, 200433 China

**Keywords:** Astrocytes, Reactive astrocyte, Astrogliosis, CNS injury, Repair

## Abstract

In the mammalian central nervous system (CNS), astrocytes are the ubiquitous glial cells that have complex morphological and molecular characteristics. These fascinating cells play essential neurosupportive and homeostatic roles in the healthy CNS and undergo morphological, molecular, and functional changes to adopt so-called ‘reactive’ states in response to CNS injury or disease. In recent years, interest in astrocyte research has increased dramatically and some new biological features and roles of astrocytes in physiological and pathological conditions have been discovered thanks to technological advances. Here, we will review and discuss the well-established and emerging astroglial biology and functions, with emphasis on their potential as therapeutic targets for CNS injury, including traumatic and ischemic injury. This review article will highlight the importance of astrocytes in the neuropathological process and repair of CNS injury.

## Introduction

Although neurons have long been thought to be the central actors in the nervous system, it is vital to remember that the brain also contains a considerable number of glial cells. Glia was originally defined as non-neuronal cells with solely supporting functions, as implied by the word ‘*Nervenkitt*’ (nerve glue) [1, 2]. In recent decades, with the advancement of science and technology, we have gained a better knowledge of glial cells, and their important roles in the nervous system are attracting our attention.

Neuroanatomists first described astrocytes as one type of glia in the middle of the nineteenth century. As the largest glial population, astrocytes tile all parts of the mammalian CNS and are traditionally identified by their shape and glial fibril expression. Despite the unique partnership between astrocytes and neurons, our understanding of astrocyte biology has lagged behind that of their neuronal counterparts. Thankfully, the importance of astrocyte in CNS has recently caught our attention. Astrocytes make extensive contact with other brain cells. Aside from providing metabolic and trophic support for neurons, astrocytes have been found to fulfill a variety of vital roles in the healthy CNS, including neuronal development, synapse formation, synaptic transmission, information processing, neurovascular coupling, blood flow regulation, and homeostasis maintenance [3–6]. Of note, the heterogeneity of astrocytes has been well documented. With the advent of new technologies to dissect astrocyte heterogeneity in-depth, astrocytes can be classified in multiple ways, including spatially, morphologically, molecularly, and functionally. Astrocyte dysfunction has been linked to numerous neurological disorders, including Rett Syndrome, Fragile X Syndrome, multiple sclerosis (MS), Alzheimer’s disease (AD), amyotrophic lateral sclerosis (ALS), and Huntington’s Disease (HD) [7, 8].

In addition to their many essential functions in a healthy CNS, astrocytes respond to different insults through a process termed reactive astrogliosis, which is involved in all forms of injury and disease response, recovery, and regeneration. The functions and roles of reactive astrocytes have been extensively studied in CNS injury, including traumatic brain injury (TBI), spinal cord injury (SCI), and Ischemic stroke. Reactive astrogliosis may result in both adaptive and maladaptive effects mainly depending on its time course and dynamic features [9]. However, it remains largely unknown how these functions of reactive astrocytes change in response to CNS injury. Reactive astrocytes are heterogeneous and regulated by different molecular signals in a context-specific manner [10]. Understanding the mechanisms that regulate astrocyte heterogeneity/plasticity may have significant implications for the therapeutic targeting of specific astrocyte subsets involved in the pathogenesis of CNS injury.

This review article starts with a detailed description of the developmental origin and biological properties of astrocytes. Their functions in physiological and pathological conditions are sequentially discussed, highlighting their potential as therapeutic targets for CNS injury, including traumatic injury and ischemic stroke. The function and therapeutic value of astrocytes in other neurological diseases such as Huntington’s disease (HD), Alzheimer’s disease (AD), Parkinson’s disease (PD), and amyotrophic lateral sclerosis (ALS), please see recently published excellent reviews [11–13] for further details and references.

## Astrocyte Development and Biology

### Astrogenesis

**Fig. 1:**
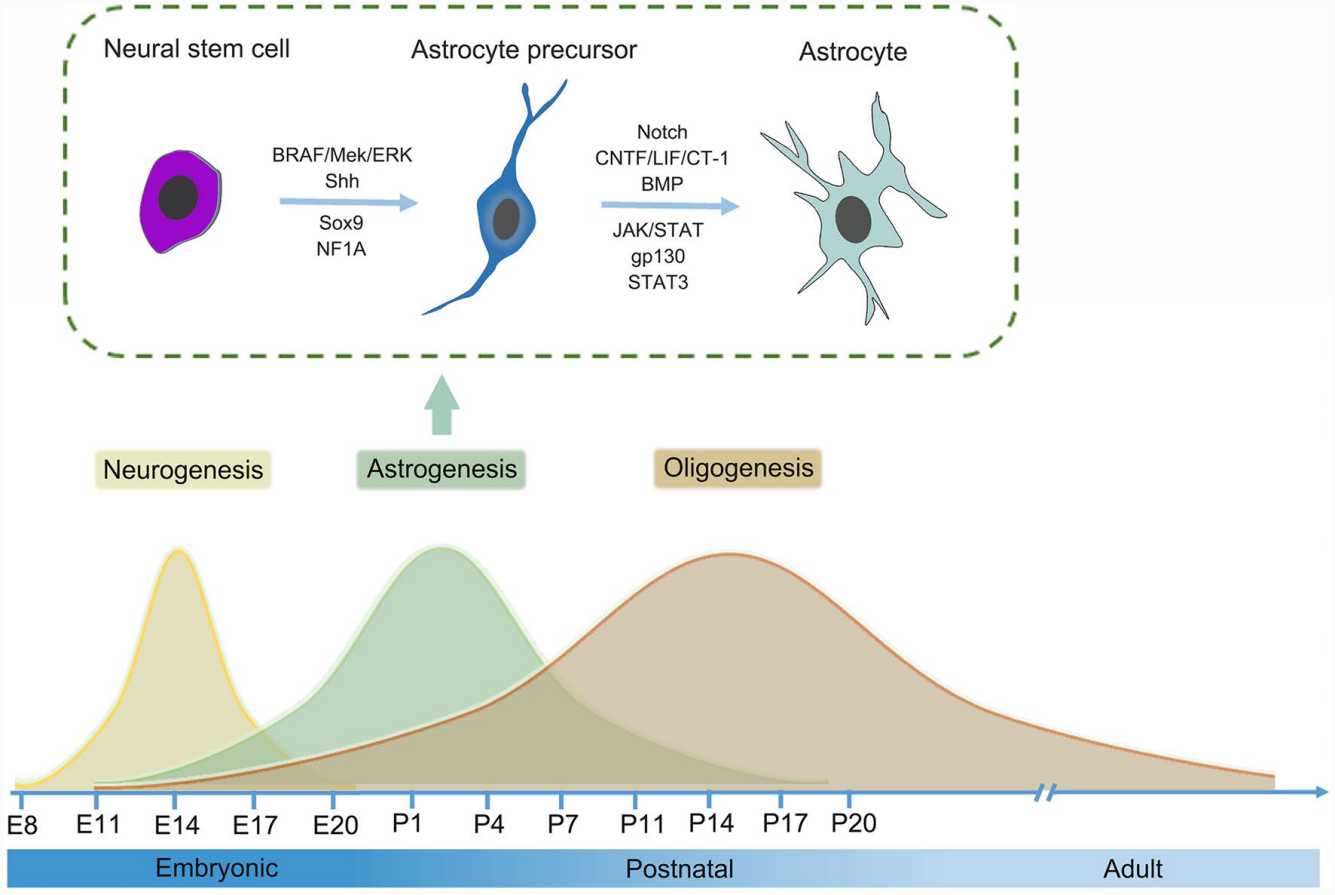
Graphical overview of astrocyte development. The diagram summarizes the developmental timelines of neurons and glial cells in rodents. Note that astrogenesis peaks at postnatal day 2 (P2). Astrocytes can be generated from a variety of sources, including perinatal radial glia, embryonic glia-restricted progenitors in the ventricular zone, and postnatal and adult glia-restricted progenitors derived from transit amplifiers. The specification and differentiation of astrocyte lineage are regulated by some key factors and signaling pathways.

In mammals, astrocytes arise from radial glia and their progeny. Astrogenesis starts late in embryonic development and continues throughout the neonatal and postnatal period (Fig. 1). Radial glia in the embryonic ventricular zone (VZ), progenitors in the postnatal SVZ, and a potential third lineage derived from glial-restricted precursors are the three different sources from which astrocytes are produced in the cerebral cortex [14]. During development, radial glia arise from the early transition of neuroepithelial cells in the VZ and they are neural progenitors for both neurons and astrocytes [15]. After serving as a scaffold for neuron migration, radial glia contract their process in the perinatal period and transform into star-shaped astrocytes [16]. Then, these cells function as intermediate astrocyte precursors and migrate radially from their ventricular zone of origin to occupy restricted spatial domains in the mouse CNS, where they begin to express canonical astrocyte markers and initiate terminal differentiation to become mature astrocytes [17]. It is worth noting that the local proliferation of differentiated glia is a major astrocyte source in the postnatal cortex in mice [18].

Astroglial generation is characterized by rapid induction of glial fibrillary acidic protein (GFAP), a major component of glial fibrils [19]. GFAP continues to be expressed in mature astrocytes and serves as the specific astroglial marker. However, mounting data indicates that not all astrocytes in CNS can be represented by GFAP^+^ cells. For example, GFAP is also expressed by progenitor cells. Therefore, additional specific markers, including Aldoc (Aldolase C), S100β, GLT-1 and GLAST, and Aldh1l1 (aldehydedehydrogenase 1 family member L1) have been found to identify astrocytes [7, 20, 21]. Investigators have also taken advantage of these marker molecules to develop transgenic mice that can be used for the visualization and study of astrocytes in vivo (Table 1).

**Table 1:** Genetically tracing mice for astrocytes

Promoter	Tracing mice	References
GFAP	mGFAP-eGFP mice hGFAP-eGFP mice hGFAP-lacZ mice	[301–306]
S100β	S100β-eGFP mice S100ß-DsRed mice	[302, 307–309]
GLT-1	GLT-1-eGFP mice	[310, 311]
GLAST	GLAST-DsRed mice	[310, 311]
AldoC	AldoC-lacZ mice	[312]
Aldh1l1	Adlh1l1-eGFP mice Aldh1l1-tdTomato mice	[43, 313]
FGFR3	FGFR3-YFP mice	[122]
Cx30	Cx30-lacZ mice	[314]
BLBP	BLBP-GFP mice BLBP-eGFP mice BLBP-eYFP mice	[315–320]

GFAP: glial fibrillary acidic protein, GLT-1: glutamate transporter 1, GLAST: glutamate-aspartate transporter, Aldoc: aldolase C, Adlh1l1: aldehyde dehydrogenase 1 family, member L1, FGFR3: fibroblastic growth factor receptor 3, BLBP: brain lipid-binding, Cx30: connexin 30.

### Mechanisms Underlying Astrogenesis

Astrogenesis involves a complex interaction of intrinsic and extrinsic cellular signals. These signals act on NSCs and precursor populations to guide them into astrocyte lineage. In mammals, most of the current evidence for specific transcription factors that regulate astrocyte development comes from studies of the developing spinal cord. For example, in the ventral spinal cord, the transcription factors SOX9 and NFIA form a transcriptional cascade to induce the initiation of gliogenesis [22–24]. Sox9 and NFIA subsequently coordinate a transcriptional regulatory cascade to regulate astrocyte precursor migration and metabolism, both of which are important for astrocyte lineage development [23]. In addition, many signaling pathways have been reported to regulate GFAP, among which CNTF/LIF/CT-1 cytokine signaling via gp130 and JAK/STAT has been proven to be a significant regulator of GFAP expression and astrocyte differentiation [25–28]. Newborn neuron-derived bone morphogenetic protein (BMP)- and Notch- signaling is also implicated in regulating the expression of GFAP and some other aspects of astrocyte development [25, 29–31].

Although the development of astrocytes, including origin, initiation, and maturation, is a more complex process than that of their neuronal counterparts, several breakthroughs in this subject have significantly advanced our understanding of astrogliogenesis [32]. For example, using single-cell/single-nucleus RNA sequencing (scRNA-seq/snRNA-seq) to analyze over 298,000 cells and nuclei during macroglia (astrocytes and oligodendrocytes) differentiation from mouse embryonic and human-induced pluripotent stem cells, Frazel *et al.* computationally identified candidate genes involved in the fate specification of glia in both species and reported heterogeneous expression of astrocyte surface markers across differentiating cells. They also uncovered potential genomic regulatory sites that mediate glial differentiation by multi-omic, dual single-nuclei (sn)RNA-seq/snATAC-seq analysis [33]. Voss *et al.* used published transcriptomic data to identify five ligands, TGF-β2, NLGN1, TSLP, DKK1, and BMP4, which synergistically drive human astrogenesis. The combinatorial effects of these five ligands converge in part on the mTORC1 signaling pathway, resulting in transcriptomic and morphological features of astrocyte development [34]. However, our understanding of astrocyte development and underlying signaling mechanisms remains limited. Future research should focus on identifying reliable stage-specific astrocyte markers as well as in vivo sources of critical astrocyte-promoting signaling molecules during development. Furthermore, strategies for selectively manipulating genes that do not affect neurogenesis should also be developed.

### Characterization of Astrocytes

Astroglia was once viewed as a homogeneous cell type in the CNS. However, increasing evidence indicates that astrocytes are a heterogeneous population that exhibits great variation in many dimensions, including species, embryonic origin, shape, gene expression profile, physiological features, and function [20, 35, 36]. Astrocyte heterogeneity manifests itself in a spatio-temporal manner. For example, astrocytes have traditionally been classified into protoplasmic and fibrous astrocytes that occupy the grey matter (GM) and the white matter (WM), respectively. In the cerebellum, there are several morphologically distinct types of astrocytes described by Cajal. By performing fate mapping in primary human tissue, Allen and colleagues showed that cortical plate astrocytes originate from VZ progenitors and proliferate locally, whereas putative white matter astrocytes are morphologically diverse and originate from both VZ and OSVZ (outer subventricular zones) progenitors [17]. There are significant molecular differences between VZ-derived cortical plate astrocytes and OSVZ-derived white matter astrocytes that persist into adulthood [17]. In mice, astrocytes in different regions of the CNS exhibit molecular heterogeneity. The molecular identity of astrocytes is influenced by the tissue microenvironments of these regions, resulting in region-specific variations in gene expression and morphological complexity. For instance, cerebellar astrocytes are the most elongated, whereas astrocytes in the motor cortex have smaller territory sizes than astrocytes in the striatum [37, 38]. Of note, astrocytes display even greater diversity in their functional roles. For example, astrocytes show diverse Ca^2+^ signals within subcompartments of astrocytes and between different brain areas [39, 40].

Recently, RNAseq, proteomic analyses, and flow cytometry have been used to investigate astrocyte heterogeneity [41, 42]. Genetic approaches, such as translational ribosome affinity purification, promoter fragment labeling, and interceptional strategy, have also been adopted to access specific astrocyte subpopulations from intact tissues [41–45]. For example, using an intersectional fluorescence-activated cell sorting–based strategy, the Deneen group identified distinct astrocyte subpopulations A, B, C, D, and E from the olfactory bulb, cortex, brainstem, thalamus, and cerebellum that show extensive molecular diversity [43]. Interestingly, population C astrocytes are found to be in large proportion in the cortex and differentially support synaptogenesis, while they are detected at much lower levels in the spinal cord. Batiuk and colleagues discovered five astrocyte subtypes throughout mouse forebrain regions using single-cell RNA sequencing [46]. Pioneering research employing genetic and lineage-tracing methods indicated that molecularly diverse subtypes of astrocytes arise from various areas in the developing spinal cord [47, 48]. Importantly, Zhuang group provided a molecularly defined and spatially resolved cell atlas of the whole mouse brain in which they observed a high diversity of astrocytes, including 36 cell clusters with unique spatial distributions [49]. Of note, many differences have been confirmed between rodent and human astrocytes from development to morphological and functional characterization [50], highlighting the need to integrate animal research with human data. For example, human astrocytes are nearly 30 times the size of rodent astrocytes and extend almost 10 times as far as rodent astrocytes [51, 52]. Interestingly, evidence has shown the existence of human-specific astrocyte types endowed with distinctive shapes and diverse functions [53]. Overall, the cellular properties of astrocyte heterogeneity, and how the heterogeneous astrocytes monitor their diverse roles, are only beginning to be recognized.

## Astroglial Functions in Healthy CNS

**Fig. 2:**
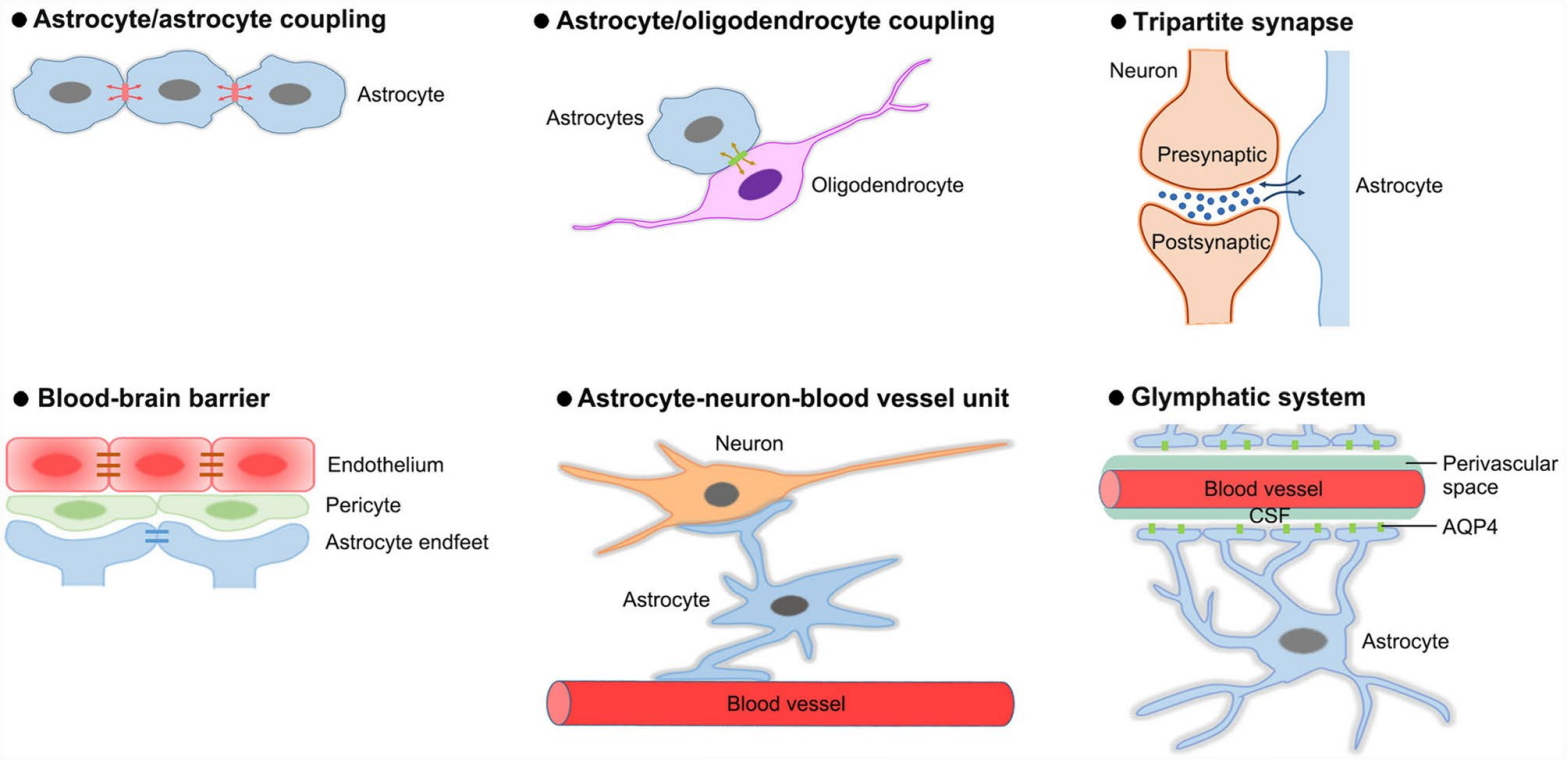
Astrocytes contribute to forming some special functional structures. These functional structures, including astrocyte/astrocyte coupling, astrocyte/oligodendrocyte coupling, tripartite synapse, blood-brain barrier, astrocyte-neuron-blood vessel unit, and glymphatic system, provide the structural basis for intercellular crosstalk of astrocytes with themselves or other cell types.

As a non-neuronal glial cell type, astrocytes lack axons and the ability to form action potentials and were traditionally considered to only provide structural, trophic, and metabolic support for neurons [1, 2]. It is worth noting that astrocytes are active communication elements of CNS that have extensive connections with other cells and tissues, including vasculature, neurons, oligodendrocytes, and microglia, and they can release a variety of regulatory signals (e.g. gliotransmitters) and form some special functional structures (e.g. tripartite synapse, blood-brain barrier and neurovascular unit) (Fig. 2). Therefore, astrocytes are now widely accepted as being essential in neural development and the maintenance of normal physiological functions of the CNS.

### Maintaining CNS Homeostasis

Mature astrocytes play essential roles in the key processes relevant to CNS homeostasis including neurotransmitter recycling, ionic balance, and BBB maintenance. It has been well documented that astrocytes contribute to maintaining extracellular homeostasis of neuroactive substances including K^+^, H^+^, glutamate, and GABA. Astrocytes take up excess extracellular K^+^ ions by expressing K^+^ channels, and distribute them through the gap junction-coupled astrocytic syncytium; these ions are extruded at sites of low extracellular K^+^ level [54]. Interestingly, astrocytes also discharge K^+^ straightforwardly into the circulatory system via direct release into capillaries by their endfeet connections [55]. In spite of the fact that glutamate is an important excitatory neurotransmitter in the CNS, excessive glutamate levels will trigger excitotoxicity [56]. Astrocytes were shown to express the high-affinity glutamate transporters excitatory amino acid transporters 1 and 2 (EAAT1 and EAAT2), which can take up the neurotransmitter glutamate to maintain glutamate homeostasis [57, 58]. Astrocytes also express a high density of high-affinity GABA transporters to take up GABA and regulate its homeostasis [59, 60]. Of note, upgraded intracellular Ca2^+^ waves in astrocytes enhance the release of glutamate and GABA [61–64]. In addition, as one of the major components of the blood-brain barrier (BBB), astrocytes play essential roles in the maintenance of BBB to protect the brain from toxic substances in the blood (as discussed in ‘*Astrocyte-vascular interactions*’ section).

### Astrocyte-neuron Interaction

Astrocytes show profoundly ramified processes, with each astrocyte contacting ∼100,000 synapses in mice [65] and up to 2,000,000 in humans [51]. Bidirectional communication between astrocytes and neurons is critical for proper brain development and functions.

Astrocytes have been shown to control neuronal maturation and synaptogenesis by synthesizing extracellular matrix proteins, adhesion molecules, and trophic factors. In the CNS, astrocytes are the primary source of extracellular matrix (ECM) proteins and adhesion molecules, including laminin, N-cadherin, neural cell adhesion molecule (NCAM), and fibronectin, which can either promote or inhibit neurite outgrowth during development [66]. Astrocytes are also known to secrete growth factors such as nerve growth factor (NGF), brain-derived neurotrophic factor (BDNF), neurotrophin-3 (NT-3), and fibroblast growth factor (FGF). These molecules are involved in regulating neuronal maturation and survival [66, 67]. Astrocytes are also essential regulators of neuronal synapse formation and maturation through their capacity to connect with synapses in a dynamic and bidirectional manner [68, 69]. A number of astrocyte-secreted factors such as cholesterol and thrombospondins (TSP) family proteins have been identified to promote synaptogenesis between neurons (Table 2). For instance, astrocyte-derived TSP can induce cortical synaptogenesis in a sex-specific manner [70]. The transmembrane protein CD38 is abundantly expressed in astrocytes in the postnatal brain and is important for astroglial development and regulating synapse formation [71]. In addition, it has been demonstrated that cell adhesion molecules mediate direct contact between astrocytes and neurons, which is essential for both synaptogenesis and astrocyte morphogenesis [69]. Interestingly, astrocytes’ heterogeneity allows them to display regional-specific abilities in boosting synaptogenesis [68].

**Table 2:** Astrocyte-derived factors regulate synaptogenesis of different types of synapses

Astrocyte-derived factors	Synaptogenesis	References
Thrombospondins	Excitatory synapse formation	[319–321]
Hevin/SPARC	Excitatory and cholinergic synapse formation	[322–326]
Glypicans	Excitatory synapse formation and maturation	[327–331]
TGF-β	Excitatory and inhibitory synapse formation	[332–335]
BDNF	GABAergic synaptogenesis	[336]
Chordin-like 1	Excitatory synapse maturation	[337]

It is well known that astrocytes sense neuronal activity by activating ion channels, transporters, and receptors, leading to rapid depolarization and/or an increase in intracellular calcium [64, 72]. These alterations in astrocytes, as well as the release of glial-derived factors, can signal back to neurons and shape their synaptic transmission [73]. Based on their microenvironment, astrocytes have specialized roles in regulating neuronal activities. For instance, gray matter astrocytes wrap synaptic terminals and influence synaptic transmission, whereas white matter astrocytes contact Ranvier nodes and may modulate spike transmission. By participating in tripartite synapses, astrocytes constitute a physical barrier to diffusion, limiting the spillover of neurotransmitters both geographically and temporally and thereby decreasing extrasynaptic transmission [74, 75]. Interestingly, astrocytes were shown to regulate the level of activation of presynaptic metabotropic glutamate receptors on glutamatergic terminals, thus controlling synaptic strength at excitatory synapses [76]. In addition, astrocyte-derived ATP was reported to modify synaptic efficacy and neuronal excitability [77–79].

### Astrocyte-oligodendrocyte Interaction

Gap junctions are not only formed between astrocytes but also between astrocytes and oligodendrocytes, providing a basis for direct cell-to-cell communication by allowing ions and small molecules to pass between cells. Astrocytes and oligodendrocytes are shown to express different sets of gap junction protein connexins (Cx). Astrocytes express Cx43 and Cx30, and some of them Cx26, while oligodendrocytes express Cx47, Cx32, and Cx29 (or its ortholog Cx31.3 in humans) [80–82]. Astrocytes are coupled to oligodendrocytes via heterotypic gap junctions, primarily mediated by Cx43- Cx47 or Cx30-Cx32, depending on the anatomical region [82–84]. The astrocyte/oligodendrocyte coupling is essential for preserving ionic equilibrium and delivering energy to oligodendrocytes [85]. Based on this intercellular crosstalk, astrocytes also play key roles in oligodendrocyte survival, maturity, and myelination [86–88]. Of note, it is suggested that neurons, astrocytes, and oligodendrocytes form a neuron-astrocyte-oligodendrocyte tri-cellular compartmentation that is critical for metabolic interactions between them [89].

### Astrocyte-vascular Interaction

It is estimated that roughly 97% of astrocytes contact blood vessels [90]. Astrocyte-vascular interactions play a number of important roles in angiogenesis and the induction and maintenance of BBB [91]. When co-cultured with astrocytes, endothelial cells (ECs) are found to form capillary-like structures [92–94]. In vivo, astrocytes migrate into the developing retina before the retinal vasculature is established, suggesting that astrocytes are required for capillary angiogenesis [94–96]. Extension of the superficial vascular plexus is slowed down by genetic mutations that inhibit astrocyte migration through the malformed inner limiting membrane of the retina [97, 98]. It remains largely unknown about the mechanism underlying astrocyte-involved angiogenesis in the brain. Astrocytes are shown to induce the mitogenesis and morphogenesis of cerebral capillary ECs by releasing cytochrome P450-derived epoxyeicosatrienoic acid [99]. Of note, astrocytes can trigger angiogenesis in the brain by activating the Wnt/β-catenin pathway target genes Axin2 and Notum in ECs via astrocyte HIFa [100]. As a specialized structure between the endothelial cells of brain capillaries and the brain tissue, BBB protects the brain from toxic substances in the blood, playing a fundamental role in maintaining brain homeostasis [101]. Astrocytes use their specialized processes, the end-feet, to enwrap the periphery of the vascular structure [102]. Perivascular astrocytes interact with ECs through specialized forms of communication such as calcium signaling and extracellular vesicles (EVs) [103, 104]. The shuttle mechanism of glutamyl-cysteinyl-glycine (GSH) is also involved in astrocyte-EC interaction [105]. Interestingly, astrocytes can modulate BBB induction, including tight junction development and transport system expression [106, 107]. For instance, the tight junction proteins in the BBB are up-regulated by co-culturing with astrocytes [108, 109], while astrocyte ablation disrupts tight junction function, and the resulting BBB malfunction is difficult to restore [110]. Therefore, astrocytes are essential for BBB integrity and their loss cannot be adequately compensated for by other types of cells. Together, an understanding of how astrocytes affect CNS angiogenesis and barrier genesis is crucial for developing new interventions to treat neurovascular pathologies.

### Astrocyte-immune Cell Interaction

As tissue-resident stromal cells of the CNS, astrocytes play an increasingly important role in providing a ‘niche’ that supports and regulates tissue-resident immunity [111]. The interaction between astrocytes and microglia (CNS-resident immune cells) has been brought to our attention [111, 112]. The astrocyte-microglia interaction is partly maintained by the secretion of mediators such as growth factors, neurotransmitters, glial transmitters, cytokines, chemokines, etc. For example, astrocytes secrete interleukin (IL)-33 to enhance microglial engulfment of synaptic proteins during development, thus limiting excitatory synapse numbers [113]. Of note, cultured astrocyte-derived IL-34, transforming growth factor β (TGF-β), and cholesterol are required for the survival of ramified microglia, suggestive of important roles in the maintenance of microglial homeostatic state in CNS [114]. The meninges are a complex tissue involved in the functional regulation of CNS-resident cells [115]. For instance, meningeal natural killer (NK) cells have been recently shown to modulate astrocyte function [116]. Meningeal NK cell-derived IFN-γ promotes T cell apoptosis by inducing TNF-related apoptosis-inducing ligand (TRAIL), a pro-apoptotic molecule, on astrocytes at the edge of meninges, which can limit pathogenic autoimmune responses in the CNS. In addition to these, communications between astrocytes and other peripheral immune cells remodel the CNS microenvironment under pathological conditions (see below).

## Astroglial Response to CNS Injury

### Astrocyte Reactionary Changes

In addition to their major neurosupportive, homeostatic roles in the healthy CNS, astrocytes become reactive in response to multiple pathogenic acute or chronic insults including inflammation, neurodegeneration, and trauma-induced injuries. The astrocyte reactionary changes are defined as reactive astrogliosis [117–119]. Here, our discussion will focus on reactive astrogliosis primarily triggered by CNS injury, including traumatic and ischemic injury.

**Fig. 3:**
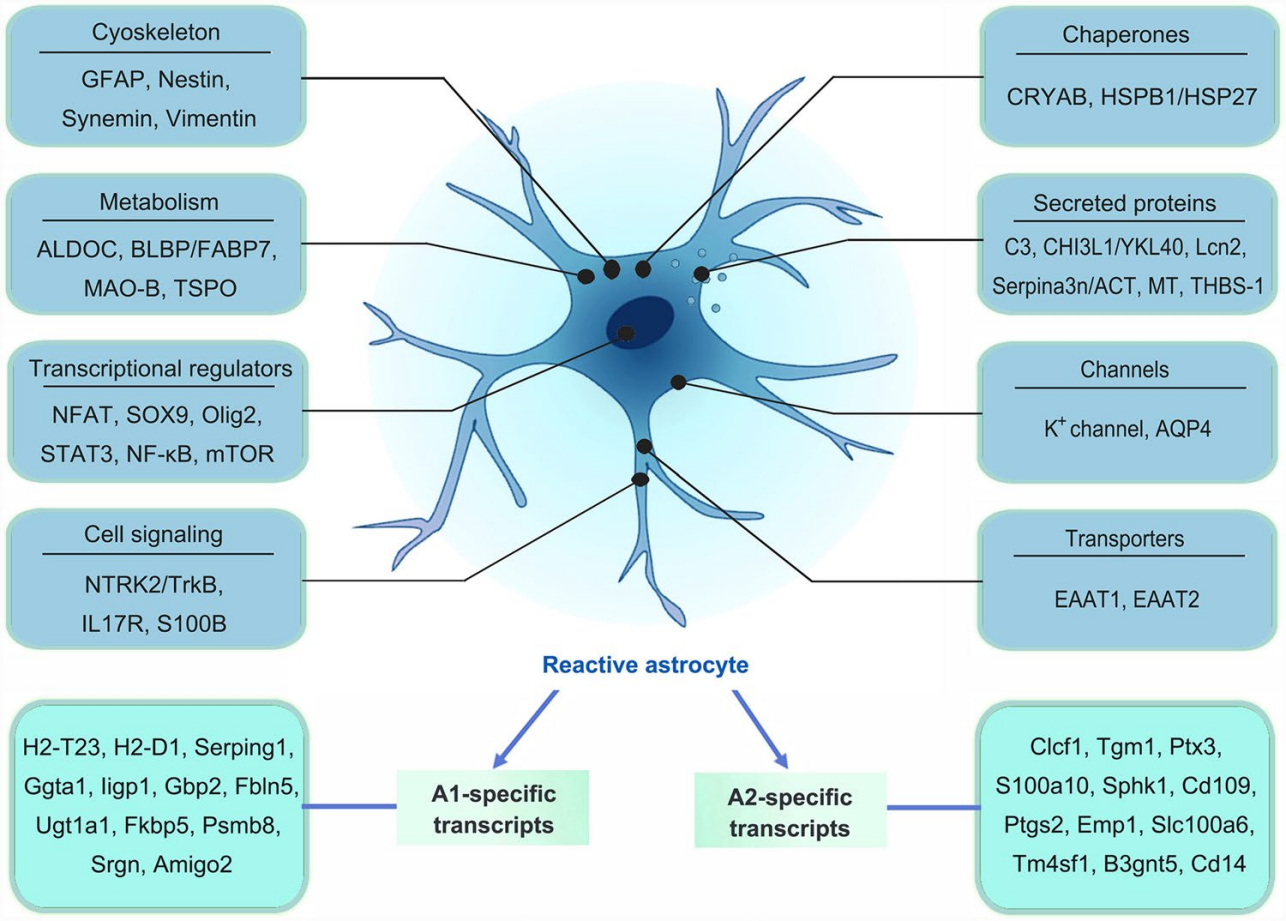
Genetic and molecular changes in reactive astrocytes. In response to CNS insults, reactive astrocytes produce many types of intercellular signaling molecules, including cytokines, chemokines, energy metabolism molecules, toxic amino acids, or intracellular signaling molecules including ions and receptors through gene expression. These molecules may be potential markers for reactive astrocytes. Note the specific transcripts expressed by A1 and A2 reactive astrocytes. The list is not meant to be exhaustive; other markers may exist and more may be added over time.

Following traumatic CNS injury, resident astrocytes around the lesion site undergo a finely graded continuum of biochemical, morphological, metabolic, and physiological remodeling [118]. These astrocytes adopt a so-called ‘reactive’ state, resulting in a change of their function or alteration of the balance between their neurosupportive and neurotoxic features. Reactive gliosis is accompanied by upregulated gene and protein expression, among which GFAP, a major protein component of astrocyte intermediate filaments, is a sensitive indicator for reactive astrocytes that is widely used (Fig. 3). The upregulation of GFAP mRNA and protein is an early response to injury, which is often parallel to the severity of the injury [120]. Of note, GFAP is also expressed by progenitors, depending on the developmental stage [121, 122]. Moreover, physiological stimuli such as physical activity also influence GFAP expression [123]. Therefore, GFAP expression alterations may indicate physiological adaptive plasticity rather than just a reflexive reaction to pathogenic stimuli. It should be noted that the expression of all or part of pre-determined relevant molecular markers is not sufficient by itself to define the functional phenotype of reactive astrocytes. In the process of reactive astrogliosis, the increased expression of GFAP in astrocytes is largely mirrored by the changes in the cytoskeleton, with hypertrophy and process extension. These reactive astrocytes re-enter the cell cycle and migrate centripetally to the lesion site, ultimately converted into scar-forming astrocytes and forming a scar border around the lesion core [124, 125]. The detailed progression of reactive gliosis and astroglial phenotypic changes was investigated following stab wound-induced TBI in mice in a recent study [126]. In addition, energy metabolism switching during reactive astrogliosis has attracted our attention. Astrocytes are at the center of energy regulation in the CNS. For instance, glycogen, the brain's main form of energy storage, is almost entirely located in astrocytes [127]. Astrocytes have a full capacity for glucose metabolism, including all glucose metabolic processes, and they transport lactate to neurons through the astrocyte-neuron lactate shuttle to support the high energy expenditure of neurons [128]. Because activation of astrocytes requires enhanced protein synthesis and trafficking, when astrocytes transition from the 'quiescent' to the 'reactive' state, they may undergo or be accompanied by metabolic switching to fulfill their energy demands, as well as functional changes in response to injury [129]. For example, increased glycolysis can accelerate inflammation by fueling the astrocyte activation [130, 131]. Importantly, the upregulated glycolysis in astrocytes is critical not only for their survival but also for the regulation of brain activation after injury [132–135]. Therefore, deciphering the metabolic properties of reactive astrocytes will expand our understanding of the biological processes of reactive astrogliosis, and may shed light on the potential therapeutic targets for CNS injury.

### Mechanisms Underlying Reactive Astrogliosis

**Fig. 4:**
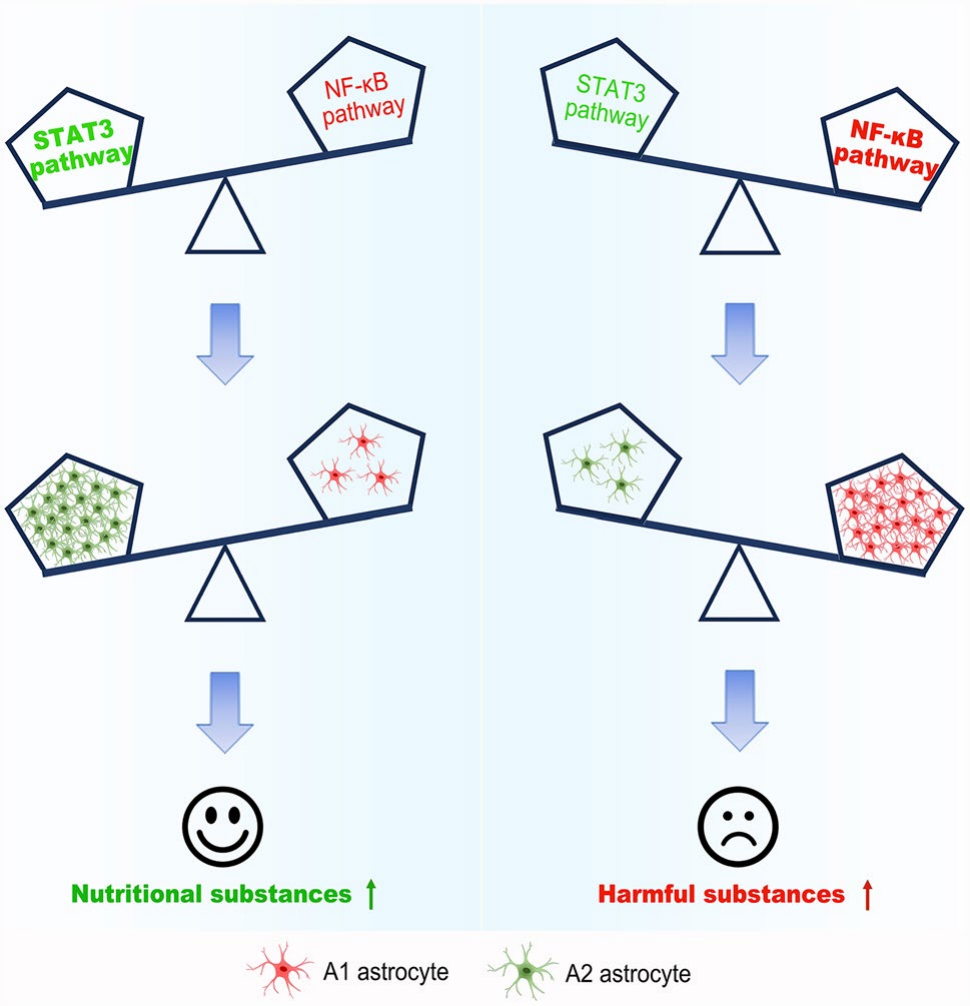
A1 vs. A2 reactive astrocytes. At present, it is suggested that astrocytes are transformed into A1 reactive astrocytes via the NF-κB pathway or into A2 reactive astrocytes via the STAT3 pathway. The A1 and A2 reactive astrocytes produce harmful and nutritional substances, respectively, which mediate maladaptive and adaptive effects.

As far, as the cellular and molecular mechanisms that control astrocyte reactivity, scar formation and maintenance are not fully understood. Increasing amounts of studies have identified many signaling pathways and molecules that modulate astrocyte reactivity, such as signal transducer and activator of transcription 3 (STAT3), nuclear factor-kappa B (NF-κB), and nuclear factor of activated T-cells (NFAT). During astrogliogenesis, STAT3 is expressed in the brain and involved in the regulation of CNS development [136, 137]. Induction of gp130-related cytokines, interleukin-6 (IL-6), leukemia inhibitory factor (LIF), and oncostatin M (OSM) can activate the Janus kinase (JAK)-STAT3 signaling pathway and up-regulate GFAP expression in reactive astrocytes [138]. In acute CNS injury, the JAK-STAT3 pathway plays a critical role in causing polarization of astrocytes, controlling astrocyte reactivity, and glial scar formation [139, 140]. MiR-21 is differentially regulated by BMP signaling pathways and modulates reactive astrocyte hypertrophy both in vitro  [141] and in vivo  [142]. Dicer1 and miR-17-5p are required for reactive astrocyte proliferation via the JAK-STAT3 pathway after SCI in the mouse [143]. NF-κB is also an important pathway involved in reactive astrogliosis, which is induced by pro-inflammatory mediators, including cytokines (TNF, IL-1β, and IL-17), reactive oxygen species (ROS), Toll-like receptor (TLR) agonists and environmental factors [144–147]. Activation of the NF-κB pathway in astrocytes results in pathological changes in the CNS, while down-regulation of the NF-κB pathway in astrocytes is associated with a reduction in CNS inflammation or injury in a variety of conditions, including SCI [148–150]. Interestingly, based on bioinformatics analysis of hippocampal proteomics, the NF-κB pathway is also confirmed to play critical roles in lead exposure-induced activation of astrocytes [151]. Of note, activation of the NF-κB pathway may result in an inflammatory environment that is not conducive to neural regeneration and repair. However, activation of the STAT3 pathway, in contrast to the NF-κB pathway, generally performs various tasks that contribute to cell survival and lesion repair (Fig. 4). In addition, calcineurin-NFAT signaling has also been shown to regulate reactive astrogliosis. Calcineurin, a serine/threonine-protein phosphatase, can be activated by elevated intracellular Ca2^+^ and subsequently binds to and dephosphorylates NFAT, resulting in nuclear translocation and transcriptional activation of NFAT [152]. After CNS injury, the calcineurin-NFAT pathway is activated and triggers reactive/inflammatory processes in astrocytes [153–155]. Selective blockade of calcineurin-NFAT signaling reduces astrocyte activation and increases synaptic functional recovery after acute CNS injury, suggestive of a key mechanism for reactive astrogliosis and CNS repair [155]. After CNS injury, reactive astrocytes have the capacity to re-enter the cell cycle, which is also an important feature of reactive astrogliosis. There are a number of molecular factors involved in regulating the proliferation of reactive astrocytes, including Shh signaling [156], Cdc42 [157], endothelin-1 [158], Galectin 1 and 3 [159], and FGF signaling [160].

It is worth mentioning that reactive astrogliosis is reversible under certain conditions, and that the local environment plays a decisive role in the fate of the astrocyte in situ. For example, the environment-dependent plasticity of reactive astrogliosis was well demonstrated in a recent study [161]. Naïve astrocytes exhibited the properties and marker gene expression of reactive astrocytes at 7 days and of scarring astrocytes at 14 days after being transplanted into the conclusively injured spinal cord, while they remained unchanged in morphology and gene expression profile when transplanted into the uninjured spinal cord. In addition, reactive astrocytes isolated from the injured spinal cord form scarring astrocytes 7 days after transplantation into the injured spinal cord, but revert in retrograde to naive astrocytes when transplanted into the naive spinal cord. Up-regulation of type 1 collagen (Col 1) and N-cadherin were observed around the lesion, and the interactions between up-regulated Col 1 and its receptor integrin β1 and the N-cadherin-mediated cell adhesion were shown to play essential roles in inducing reactive astrocytes transformation into scarring astrocytes [162].

### Heterogeneity and Roles of Reactive Astrocytes

In recent years, the heterogeneity of glial cells has attracted much attention. As highly plastic cells, astrocytes response to diverse forms of CNS injury with heterogeneous changes of their phenotypes in a time, location, and context-dependent manner. These heterogeneous reactive astrocytes may be either neurodegenerative or neuroprotective under pathological conditions. However, the heterogeneity of reactive astrocytes remains poorly defined, thus understanding the heterogeneity and the underlying mechanisms will be important for the potential therapeutic targeting of astrocyte subsets that are involved in the pathogenesis of CNS injury.

Reactive astrogliosis is a range of underlying molecular, cellular, and functional changes in astrocytes that vary in response to various forms and severity of CNS injury. Astrocyte responses are well addressed in animal models of focal CNS insult such as stab wound injury and controlled cortical impact (CCI). After a stab wound to the brain, heterogeneous reactive astrocytes are observed, including GFAP^+^ astrocytes, GFAP^+^ hypertrophic astrocytes, proliferating astrocytes, and atypical astrocytes, which form a glial border around the injury site [163–165]. Interestingly, astrocytes nearest to the lesion take on a palisading character and are more likely to proliferate. Together with other glia, these palisading astrocytes form a border to separate undamaged brain tissue from damaged areas. Clinically, most traumatic CNS injuries are characterized by diffuse damage which is caused by acceleration-deceleration forces, resulting in brain tissue shearing, compression, and tension [166, 167]. Unlike focal CNS injury, which usually causes blood vessel rupture and exposes brain tissue to large blood-borne factors, diffuse CNS injury mainly results in damage to the BBB and causes leakage of small (≤10 kDa) factors. Astrocytes response to diffuse CNS injury in a ‘classical’ or ‘atypical’ manner [165, 168–170]. The classically activated astrocytes become reactive with up-regulation of intermediate filaments such as GFAP and vimentin, and they have some hypertrophy but do not prolong their processes. The atypically activated astrocytes rapidly lose many of the homeostatic proteins (Glt1, Kir4.1, glutamine synthetase) and housekeeping proteins (GAPDH, S100b), but without obvious up-regulation of GFAP or other intermediate filaments. Of note, both classically and atypically activated astrocytes do not proliferate in response to diffuse CNS injury, possibly due to a lack of factors that initiate proliferation [171]. Interestingly, the heterogeneous morphological changes of reactive astrocytes are also dependent on the degree of stimulation [172]. When the CNS damage is mild, the processes of reactive astrocytes expand, accompanied by process and cell body hypertrophy, and reactive astrocytes are further hypertrophy and overlapped in severe CNS injury. Within the severely injured CNS, tissue can be reorganized to a considerable extent, so they are less likely to return to normal structure [6].

The location of astrocytes and the time of injury are also key factors affecting the reactive astrocyte heterogeneity. For example, as the distance from the lesion center increases, the morphology of reactive astrocytes changes. When reactive astrocytes are far from the lesion center, they have a stellate morphology and typical hypertrophy process and their protrusions have no preferential orientation. Reactive astrocytes close to the damaged core, on the other hand, exhibit a prolonged process that is directed toward the lesion center. Interestingly, more than a third of astrocytes around the lesion site re-enter the cell cycle after stab wound-induced TBI in the cortical grey matter [173–175]. However, there are considerably fewer astrocytes proliferating in white matter after a stab wound or CCI [176, 177]. In addition, heterogeneity is also observed in the proliferative response of reactive astrocytes at different time points after CNS injury. On the first day after the stab wound, there are only a few astrocytes proliferating acutely, but it rises to 25% in the first week after the stab wound [173]. Of note, reactive astrocytes cease to proliferate during the second week following focal TBI.

Astrocytes show extensive and heterogeneous changes in gene expression in response to different CNS insults and fluctuate over time during the disorder [36]. Recently, transcriptomics has been used to analyze the distinct molecular states of reactive astrocytes [159, 178–183]. These studies contribute to identifying multiple reactive astrocyte clusters and their complex functional changes, such as novel regenerative functions, proliferation, neural stem cell potential, and loss of homeostatic functions, suggestive of possible targets for therapeutic intervention. For instance, reactive astrocytes can be fundamentally divided into proliferative border-forming or non-proliferative cells in models of CNS trauma [36]. The proliferative border-forming reactive astrocytes are critical for reshaping the BBB around the lesion and for protecting and preserving adjacent functional nerve tissue. Loss or reduction of these cells will result in the spread of inflammation, increased loss of nerve tissue, and ultimately reduced functional recovery [184–187]. The non-proliferative reactive astrocytes show only variable changes in molecular expression and cellular morphology, and they may maintain functional interactions with the same cellular components that they interact with in healthy CNS [6, 179, 188]. Interestingly, transcriptional analysis indicates that astrocytes adopted an ‘A1’ neurotoxic phenotype in a mouse model that receives intraparenchymal injection of lipopolysaccharide (LPS), while they are induced to adopt an ‘A2’ neuroprotective phenotype after ischemic stroke [179]. The A1 reactive astrocytes lose most normal functions and up-regulate many genes (such as complement cascade genes) that have been previously reported to be destructive to synapses. On the contrary, the A2 reactive astrocytes up-regulate many neurotrophic factors and thrombospondins that contribute to neuronal survival and synapse repair, respectively. A follow-up study shows that the A1 reactive astrocytes are induced by IL-1α, TNF-α, and C1q derived from LPS-activated microglia [189]. TBI-induced inflammatory and reactive astrocyte phenotype can be partially blocked by microglial depletion. Using single-cell transcriptomic analysis, Cheng's group identified 5 astrocytic subpopulations (clusters 1-5) in the spinal cord after SCI [183]. Clusters 1-3 astrocytes are also found in the uninjured spinal cord, whereas cluster 4/5 astrocytes are only found in the damaged spinal cord, expressing *Vimentin*, Gfap, and low levels of *Sox2*. Importantly, Cluster 5 astrocytes are located close to the injury epicenter and were Mki67-positive, suggesting that they are proliferating. Based on RNA sequencing, protein detection, and assay for transposase-accessible chromatin with high-throughput sequencing (ATAC-seq), Sofroniew group identified more than 12,000 differentially expressed genes (DEGs) that are potentially associated with astrocyte reactivity across diverse CNS disorders in mice and humans [182]. These DEGs show significant heterogeneity across disorders, and the DEG diversity is determined by combinatorial, context-specific interactions between transcriptional regulators. In many cases, it is worth noting that transcriptomic changes are observed among reactive astrocytes that do not demonstrate substantial differences in other aspects such as cell morphology and proliferation. It is suggested that future classification of reactive astrocytes should take into consideration several types of information concerning astrocyte shape, cell lineage, proliferative state, molecular expression, cellular interactions, and functions.

Overall, reactive astrocytes are highly heterogeneous, which has spurred speculation about the possible existence of multiple astrocyte subtypes with different functions. However, the underlying mechanisms that determine the astrocytic heterogeneity remain largely unknown. Whether the astrocyte reactivity is affected by baseline astrocyte heterogeneity is also an open question.

### Immune Response of Reactive Astrocytes

A growing body of evidence suggests that reactive astrocytes are an important component of multicellular CNS immunity. Many receptors for pathogen-associated molecular patterns (PAMPs) and damage- or danger-associated molecular patterns (DAMPs) that are known to induce immune responses are expressed on astrocytes, including Toll-like receptors and purinergic P2 receptors [190, 191]. Therefore, astrocytes have been thought to respond to multiple activators of immune responses, such as microbial pathogens, environmental toxins, and tissue damage [36, 144, 192–194]. For example, extracellular ATP can regulate immune responses by binding to purinergic P2 receptors on astrocytes, and this ATP-mediated purinergic signaling is a key regulator for damage-associated molecular patterns or alarmins [191]. Under pathological conditions, damaged cells-derived extracellular ATP activates P2X7 receptors on astrocytes and induces the production of chemokines, cytokines, and reactive oxygen species, triggering pro-inflammatory responses [195, 196]. Activation of astrocyte P2X7 receptors also initiates the nucleotide-binding and oligomerization domain-like receptor family pyrin domain-containing protein 3, an essential inflammasome regulator [197]. Microglia-derived ATP can trigger P2Y1 receptor activation in astrocytes, which induces glutamate release and increases excitatory neurotransmission [198].

Astrocytes are also capable of responding to a wide range of cytokines, chemokines, growth factors, and other molecules that mediate intercellular communication during immune responses [36]. For instance, astrocytes interact directly with microglia via exposure to specific cytokines, and their interactions can have both beneficial and maladaptive effects on the immune response [199]. The most well-known is that LPS-activated microglia instruct astrocytes into an ‘A1” neurotoxic reactive subtype [189]. On the contrary, astrocytes can counteract the pro-inflammatory effects of sphingosine 1-phosphate (S1P) in microglia by releasing the γ-subunit of complement 8, which interacts with S1P receptor 2 [200].

Phagocytosis has traditionally been done by specialized phagocytes, the microglia in the CNS. However, there is growing evidence that astrocytes, non-professional phagocytes, also contribute to the phagocytic process [201–203]. For example, astrocyte-mediated phagocytosis was reported to clear dead cells and parts of live cells, such as synapses and axons [204, 205]. Gene profiling studies indicate that astrocytes express genes enriched in engulfment pathways such as TAM receptors, draper/Megf10, and ABCA1 [122, 206]. The underlying mechanism of astrocyte-mediated phagocytosis please see recently published excellent papers [203, 207–210] for further details and references. Of note, astrocytes and microglia share common phagocytic targets and receptors; however, the difference or coordination between astrocytes and microglia remains elusive. After brain injury, disintegrated axons and neurons are also observed in reactive astrocytes, suggestive their phagocytic function [211, 212]. Moreover, reactive astrocytes play an essential role in myelin phagocytosis after ischemic injury [208, 213].

Taken together, reactive astrocyte-mediated immune response significantly contributes to the remodeling of damaged tissues and microenvironment, which may play an important role in the repair of CNS injury.

### Lineage Potential of Reactive Astrocytes

In the past two decades, there has been interest in the stem cell characteristics of astrocytes [214, 215]. Radial glial cells are the first glia to appear during neural development. With the commencement of neurogenesis, radial glial cells differentiate from neuroepithelial cells by gaining glial hallmarks, including Vimentin, GFAP, and glutamine synthase (GS). In the adult brain, a subpopulation of SVZ astrocytes (GFAP^+^/S100β^-^) was identified to exhibit stem cell characteristics such as multipotency and self-renewal in rodents and humans [216, 217], which has challenged the traditional definition of astrocyte as well as its functions. These adult SVZ astrocytes resemble radial glial cells, and they differentiate into transit-amplifying progenitors that generate neuroblasts. The SVZ astrocyte-derived neuroblasts mainly migrate to the olfactory bulb where they differentiate into interneurons [218, 219]. In addition, SVZ astrocytes may also act as an instructor of neurogenesis [220].

Unlike SVZ astrocytes, parenchyma astrocytes of non-neurogenic regions do not proliferate and have no NSC potential in normal healthy brains [156]. However, a proportion of reactive astrocytes in the vicinity of lesion site start to proliferate after invasive CNS injury, and some of these cells even express NSC marker nestin, suggesting that they may be reactivated to regain stem cell features [188, 221–223]. When cultured under neurosphere conditions, these reactive astrocytes show stem cell potential with self-renewal and multipotency [156, 174, 224, 225]. Further study indicates that Sonic hedgehog (SHH) signaling is necessary and sufficient to elicit the stem cell response of reactive astrocytes both *in vitro* and *in vivo* [156]. Interestingly, astrocytes in the postnatal and adult CNS can be manipulated *in vitro* and, following brain damage, *in vivo* to undergo the initial phases of neurogenesis [226–229]. For example, cultured postnatal astrocytes can be reverted into stem cells by reactivating the proper genetic program [230]. In particular, the proneural genes are capable of inducing functional reprogramming of astroglia toward a neuronal identity [231–234]. These discoveries are highly interesting because they open up the possibility that reactive astrocytes, which have been shown to return to an immature phenotype, might be converted into newborn neurons for repairing the injured brain.

## Astrocyte-targeting Therapies for CNS Injury

**Fig. 5:**
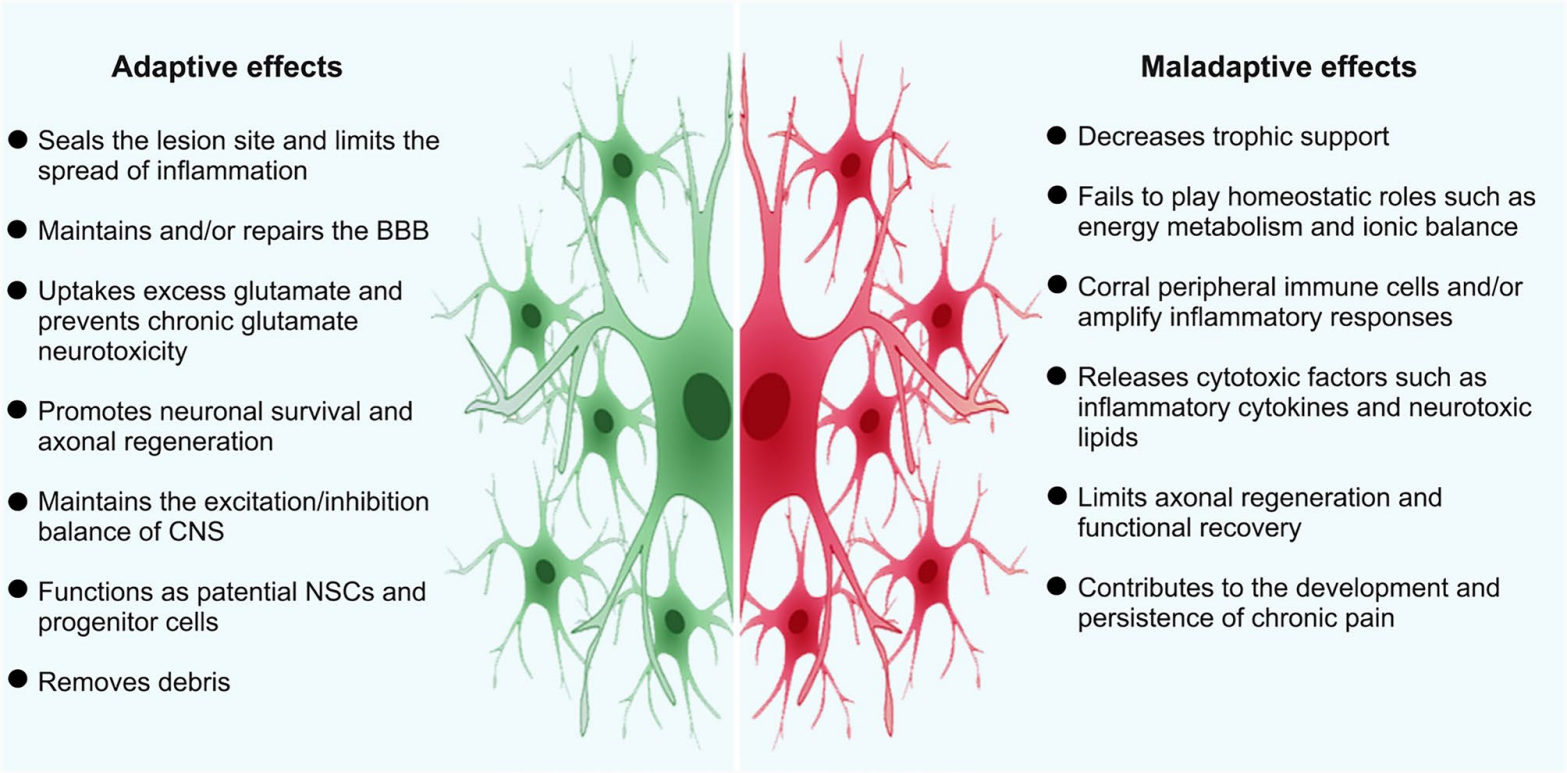
Adaptive and maladaptive influence of reactive astrogliosis/astrocytes. The role of reactive astrocytes varies substantially depending on different situations. The left- and right-hand side show the adaptive and maladaptive effects of reactive astrocytes on CNS repair after injury.

As mentioned above, astrocytes respond to CNS injury by changing their molecular make-up and cell biology, ultimately leading to alterations in astrocyte function. These alterations can be beneficial or detrimental to CNS repairing (Fig. 5). Therefore, one goal of research on reactive astrogliosis is to translate the vast amount of functional and molecular data described in the previous sections into astrocyte-targeting therapies for CNS injury.

### Traumatic CNS Injury

Traumatic CNS injuries, including TBI and SCI, are major causes of permanent physical disability and long-term neuropsychiatric impairment, and their treatment remains a significant challenge. Astrocytes are critical players in the CNS injury response in the manner of reactive astrogliosis and scar formation. As discussed in previous sections, a growing body of studies has improved our understanding of the signaling pathways and molecular mediators that trigger and regulate reactive astrogliosis and scarring. Therefore, efficiently targeting these mechanisms will harness their potential for repair and regeneration after traumatic CNS injury. For example, our group showed that triptolide, one of the major active components of the traditional Chinese herb *Tripterygium wilfordii Hook F*, could inhibit reactive astrogliosis and inflammation by blocking the JAK-STAT3 pathway and promote mouse spinal cord repair after SCI [235]. Reactive astrocyte-derived chondroitin sulfate proteoglycans (CSPGs) are considered to be major inhibitors of axonal regeneration [236, 237]. Inhibition of CSPGs formation or degradation of CSPGs by chondroitinase ABC (chABCs) results in axonal sprouting and functional recovery after SCI [238–240]. Activation of NF-κB pathway in reactive astrocytes usually induces a pro-inflammatory response. Selective inhibition of NF-κB in astrocytes has been reported to ameliorate inflammation and improve the rate of recovery after SCI [148]. BMP and endothelin-1 (ET-1) up-regulated in reactive astrocytes are the main factors that inhibit the differentiation and remyelination of OPCs, and blocking BMP and ET-1 provides a promising therapeutic strategy for increasing OPC differentiation and remyelination after SCI [241, 242]. At the sub-acute/chronic stage of traumatic CNS injury, reactive astrocytes form a prominent densely packed astrogliosis border (glial scar) surrounding the lesion epicenter, which has become a major therapeutic target in the field of SCI. The post-traumatic glial scar is traditionally regarded as a barrier to axon regeneration and functional recovery [243–245], whereas it is also reported to have beneficial effects on SCI repair [180, 243]. After a stab wound, selective ablation of scar-forming, proliferative reactive astrocytes by STAT3 deletion leads to increased inflammation and lesion volume, failure of BBB repair, and reduced motor recovery [140, 246, 247]. Using loss-of-function manipulations, we and other groups also showed that astrocyte inhibition of scar formation resulted in the failure of spontaneous axon regrowth and functional recovery after SCI [178, 185, 248]. The controversial findings about the effect of glial scar on nerve regeneration may be related to the existence of heterogeneous astrocytes. Therefore, more careful and intentional perspectives are required to develop effective strategies for modulating CNS injury-induced reactive astrogliosis or glial scar formation. Therapeutic strategies should be directed at specific aspects of reactive astrogliosis and specific molecular mechanisms for specific goals. For instance, because neurotoxic A1 reactive astrocytes are induced by microglia-derived IL-1α, TNF-α, and C1q, mice lacking IL-1α, TNF-α, and C1q survive longer than controls [249]. Of note, blocking IL-1α and TNF-α with neutralizing antibodies can inhibit A1 formation [249], and FDA-approved drugs that target IL-1α and TNF-α already exist. Interestingly, a new drug inhibiting C1q is also currently in clinical trial. As an anti-inflammatory drug acting on the NF-κB pathway, rolipram is shown to limit the expression of proinflammatory substances, including Lcn2 and iNOS, in A1 astrocytes [250].

Human and animal studies have shown that deficient astrocyte functions or loss-of-astrocytes contribute significantly to increased vulnerability to cell death for neurons, oligodendrocytes, and axons after traumatic CNS injury [251]. Therefore, given its essential functions in CNS, astrocyte transplantation has become an effective approach to replace the dysfunctional or lost astrocytes after injury and promote CNS repair [252]. For instance, transplantation of neonatal astrocytes into the damaged spinal cord can stimulate the regrowth of injured fibers and promote modest locomotor recovery after SCI [253]. Pencale *et al.* showed adult rat cortical astrocytes successfully survived, integrated, and migrated within the host parenchyma when grafted into the completely transected rat spinal cord [254]. Some scholars suggested that transplantation of glial-restricted precursors (GRPs)-derived astrocytes (GDAs) for SCI could achieve better results [255–257]. Importantly, research on human astrocyte transplantation is also being carried out gradually, which will lay a solid foundation for its clinical application [258–266]. Given the significant heterogeneity in the astrocytes, the heterogeneous astrocyte sources may have distinct effects on their differentiation, migration, and connection in the host neural network, and the implanted astrocyte activities may also be influenced by the environment of different target regions. Therefore, it is critical to select optimal astrocyte sources and appropriate injection regions in the host CNS. In addition to the transplantation of primary astrocytes, another promising approach is the transplantation of astrocytes that are genetically modified to express certain molecules, such as growth factors. These grafts act as cellular pumps to provide long-term, spatially limited administration of therapeutic molecules for CNS injury [267, 268].

As noted in previous sections, reactive astrocytes can be dedifferentiated to acquire NSC properties, providing an important new resource for endogenous repair after CNS injury [156, 225]. Although these NSC-like reactive astrocytes remain within their lineage and fail to produce neurons *in vivo* [156], their high plasticity makes them an ideal cell source amenable to fate conversion. As shown in Table 3, our and other groups have confirmed that ectopic expression of transcription factors can successfully reprogram reactive astrocytes into neurons after TBI or SCI. However, their further clinical application may be limited by the safety concerns about genetic manipulation and the technical challenges in delivering exogenous genes *in vivo*. Recently, small molecules have attracted great interest in the field of lineage reprogramming as an attractive alternative. For example, our proof-of-principle studies first provide evidence that in vivo reprogramming of reactive astrocytes into neurons can be manipulated in a chemical compound-based manner in a damaged spinal cord [269, 270]. Of note, we found that region-restrict astrocytes reveal heterogeneous susceptibility to neuronal reprogramming [271]. Therefore, the astrocyte heterogeneity should be taken into account in neuronal reprogramming, and further studies should attempt to identify the subpopulations of astrocyte subsets best suited for neuronal reprogramming.

**Table 3:** *In vivo* astrocyte-to-neuron conversion

Animal model	Source cells	Inducers	Induced cells	References
Traumatic brain injury	Adult mouse cortical astrocytes	NeuroD1	Glutamatergic neurons	[338, 339]
Traumatic brain injury	Adult mouse cortical astrocytes	Ngn2	Glutamatergic neurons	[340]
Traumatic brain injury	Adult mouse midbrain astrocytes	Ascl1	GABAergic neurons	[341]
Traumatic brain injury	Adult mouse striatal astrocytes	Sox2	Neurons	[342, 343]
Traumatic brain injury	Adult mouse neocortex astrocytes	Ngn2/Nurr1	Pyramidal neurons	[344]
Traumatic spinal cord injury	Adult mouse spinal astrocytes	Sox2	GABAergic and glutamatergic neurons	[345, 346]
Traumatic spinal cord injury	Adult mouse spinal astrocytes	DAPT	GABAergic neurons	[269]
Traumatic spinal cord injury	Adult mouse spinal astrocytes	LDN193189/ CHIR99021	GABAergic neurons	[270]
Traumatic spinal cord injury	Adult rat spinal astrocytes	Zfp521	Neurons	[347]
Ischemic brain injury	Adult rat striatal and neocortex astrocytes	Ngn2	Neurons	[348]
Ischemic brain injury	Adult mouse neocortex astrocytes	Ngn2	Neurons	[349]
Ischemic brain injury	Adult mouse cortical astrocytes	NeuroD1	Glutamatergic neurons	[288]
Ischemic brain injury	Adult primate cortical astrocytes	NeuroD1	Tbr1^+^ cortical neurons	[287]
Ischemic brain injury	Adult mouse striatal and neocortex astrocytes	Ascl1/Sox2/NeuroD1	Neurons	[350]

This table summarizes the transcription factor- and chemical compound-mediated *in vivo* astrocyte-to-neuron conversion. Note that injection operation is regarded as an injury to the brain in animal studies.

### Ischemic Stroke

Ischemic stroke is another primary cause of severe disability, and it also represents the 2nd leading cause of death worldwide [272, 273]. Despite recent breakthroughs in reperfusion procedures, treatment options for preventing progressive neuronal loss and functional impairment remain limited and mostly unsuccessful [274]. After ischemic stroke, peri-infarct astrocytes become reactive in respond to hypoxia, neuronal cell death, release of neurotransmitters, and blood extravasation by altered expression of many genes, eventually forming glial scars in the penumbra [139, 275]. At the acute stage of stroke, astrocyte activation and reactive gliosis separate the ischemic core from healthy tissue to limit tissue damage and contribute to the restoration of homeostasis. In the post-acute and chronic phases, however, reactive astrocytes operate as both positive and negative regulators of neural plasticity, such as neurogenesis, axonal sprouting, and synapse function, which is the basis for functional recovery. Therefore, the local reactive astrocytes might be appealing targets for cell-based stroke therapy [276]. Currently, astrocyte-targeted interventions, including controlling astrocyte polarization, decreasing edema, regulating glial scar formation, and reprogramming astrocytes have been shown to modulate ischemic stroke progression.

Genetic ablation of astrocyte intermediate filament proteins, such as GFAP^−/−^Vim^−/−^ mice, results in the absence of characteristic cellular hypertrophy attenuated reactive gliosis on injury [277]. Importantly, larger infarcts are observed in GFAP^−/−^Vim^−/−^ mice with ischemic stroke induced by middle cerebral artery transection, suggestive of the neuroprotective function of reactive astrocytes at the acute stage of ischemic stroke [278]. However, some negative effects of reactive gliosis have also been documented. For instance, following ischemic injury, damage-associated molecular patterns (DAMPs) released by damaged cells activate astrocyte Toll-like receptor, TNF receptor, IL-1 receptor, IFN receptor, chemokines CXCL12, CCl2, C1q, and other inflammatory signaling receptors, thereby exacerbating ischemic stroke [279]. Microglia is shown to produce TNF-α which facilitates the release of glutamate from astrocytes via SDF1a-CXCR4 signaling conductance, finally increasing neuronal excitotoxicity [280]. On the other hand, reactive astrocytes enhance the pro-inflammatory function of microglia via secreting IL-6 [281]. Therefore, regulation of these astrocyte-associated signaling pathways is an effective way to treat stroke. In addition, given the fact that astrocyte-derived extracellular vesicles (EVs) are actively involved in mediating neuroprotection and neurorepair for stroke, they are regarded as novel therapeutic targets for stroke [282]. Reactive astrocytes induced by ischemia are recently reported to participate in phagocytosis to clear neuronal debris, which is mediated by ATP-binding cassette transporter A1 (ABCA1) and the molecules in its pathway, such as multiple EGF-like-domains 10 (MEGF10) and the engulfment adapter phosphotyrosine binding domain containing 1 (GULP1) [211]. These phagocytic astrocytes may contribute to microenvironmental remodeling and neurological recovery in the ischemic penumbra.

Significant neuronal death is a common consequence of stroke, resulting in permanent brain function loss. Therefore, it is essential to replenish the missing neurons in the damaged brain area and then rebuild the functional connections between the neurons to promote post-stroke recovery. Given the lineage potential of reactive astrocytes, many researchers have been working for the past decade to program astrocytes into neurons (Table 3). For example, Notch1 signaling is reduced in astrocytes after stroke, and blocking this signaling can trigger astrocytes in the striatum and the medial cortex to undergo neurogenesis, which promotes nerve repair [283, 284]. By single-cell RNA sequencing analysis, further study shows that when these Notch1-depleted parenchymal astrocytes initiate neurogenesis, they become transcriptionally extremely similar to SVZ stem cells, progressing through an almost identical neurogenic program [285]. As a proneural factor, NeuroD1 is expressed in NSCs during the early stages of brain development, allowing the NSCs to differentiate into neurons [286]. In rodents or non-human primates with induced cerebral infarction, forced expression of NeuroD1 in astrocytes can reprogram them into functional neurons, thereby promoting neuronal repair and functional recovery after ischemic stroke [287, 288]. While this field currently faces many challenges, *in vivo* astrocyte-to-neuron conversion has attracted widespread attention as a promising way to increase the number of neurons after ischemic stroke.

### CNS Injury-related Neuropathic Pain

Neuropathic pain, an unpleasant sensory and emotional experience, is a common consequence of damage to the nervous system. For example, most SCI patients suffer from severe chronic neuropathic pain syndromes that may lead to depression, drug abuse, and suicide. Central post-stroke pain, occurring after hemorrhagic or ischemic stroke also severely affects the patient’s quality of life and rehabilitation. Of note, patients with neuropathic pain experience more severe pain and greater impairment of function and quality of life than those with non-neuropathic mechanisms [289, 290]. Although it has not yet been fully elucidated about the mechanism underlying neuropathic pain, accumulating evidence suggests that reactive astrocytes critically contribute to pain pathogenesis [291, 292]. In the spinal cord, injury-induced reactive astrocytes play a key role in the development and maintenance of central neuropathic pain by releasing various astroglial mediators to increase the activity of spinal cord nociceptive neurons [293, 294]. Following nerve damage, reactive astrocytes lose their capacity to maintain the homeostatic concentrations of extracellular potassium (K^+^) and glutamate, resulting in neuronal hyperexcitability [295]. In addition, astrocytic Cx43 is also shown to increase spinal cord synaptic transmission and sustain neuropathic pain in the late phase of SCI via releasing chemokines [293]. Spinal injection of selective Cx43 blockers can effectively reduce neuropathic pain [293]. Thus, novel strategies for the treatment of CNS injury-related neuropathic pain may be provided by altering specific signaling pathways in astrocytes.

## Concluding Remarks

Astrocytes account for approximately 20% to 40% of the total number of cells in the brain, depending on the specific brain regions and species, and they act as an active player in maintaining CNS homeostasis. Here, we review the biological characteristics and functions of astrocytes from physiology to pathology, with emphasis on their reactive responses to CNS injury. This will provide a more comprehensive understanding of astrocytes in physiological and pathological conditions and how we might ameliorate CNS injury by targeting astrocytes. However, to make astrocyte-targeting therapies possible, several fundamental questions need to be addressed. Firstly, a major challenge for astrocyte-targeting therapies is the existence of astrocyte heterogeneity and what proportion of the characterized astrocyte diversity in injured CNS represents heterogeneity vs. plasticity. Although single-cell transcriptional analysis has provided a snapshot of gene expression of the heterogeneous astrocytes, it is important to assess their functional differences and determine how to therapeutically target the specific astrocyte subsets of interest in a context-specific manner. Secondly, growing evidence indicates that astrocytes might contribute to some of the sex-difference outcomes after CNS injury [296–298], while little is known about the sex-specific effects of astrocyte changes on the repair of CNS injury. Thirdly, astrocyte-targeting therapies face the challenge of delivering drugs across the BBB. Of note, the synthetic nanoparticles have the ability to cross the BBB and thus can be used for astrocyte targeting [299, 300]. Finally, because there are key differences between the astrocytes from laboratory animals and humans, the data on astrocytes obtained from animal models should be carefully validated in humans. Given that astrocytes are difficult to obtain from patients, human-induced pluripotent stem cell (hiPSC) technological advances are being used in astrocyte research. For example, astrocytes generated from hiPSC derived from fibroblasts obtained from patients are useful for the study of CNS pathogenesis and drug screening. We believe that the resolution of these problems will increase the feasibility of astrocyte-targeting therapies.
